# A Feasibility Study to Investigate Chemogenetic Modulation of the Locus Coeruleus by Means of Single Unit Activity

**DOI:** 10.3389/fnins.2020.00162

**Published:** 2020-03-06

**Authors:** Latoya Stevens, Kristl Vonck, Lars Emil Larsen, Wouter Van Lysebettens, Charlotte Germonpré, Veerle Baekelandt, Chris Van den Haute, Evelien Carrette, Wytse Jan Wadman, Paul Boon, Robrecht Raedt

**Affiliations:** ^1^4BRAIN, Institute for Neuroscience, Department of Neurology, Ghent University, Ghent, Belgium; ^2^Laboratory for Neurobiology and Gene Therapy, Center for Molecular Medicine, Leuven Brain Institute, KU Leuven, Leuven, Belgium; ^3^Leuven Viral Vector Core, Centre for Molecular Medicine, KU Leuven, Leuven, Belgium

**Keywords:** locus coeruleus, chemogenetics, designer receptor exclusively activated by designer drugs, unit recording, clozapine, vagus nerve stimulation

## Abstract

**Aim:**

Selective chemogenetic modulation of locus coeruleus (LC) neurons would allow dedicated investigation of the role of the LC-NA pathway in brain excitability and disorders such as epilepsy. This study investigated the feasibility of an experimental set-up where chemogenetic modification of the brainstem locus coeruleus NA neurons is aimed at and followed by LC unit activity recording in response to clozapine.

**Methods:**

The LC of male Sprague-Dawley rats was injected with 10 nl of adeno-associated viral vector AAV2/7-PRSx8-hM3Dq-mCherry (*n* = 19, DREADD group) or AAV2/7-PRSx8-eGFP (*n* = 13, Controls). Three weeks later, LC unit recordings were performed in anesthetized rats. We investigated whether clozapine, a drug known to bind to modified neurons expressing hM3Dq receptors, was able to increase the LC firing rate. Baseline unit activity was recorded followed by subsequent administration of 0.01 and 0.1 mg/kg of clozapine in all rats. hM3Dq-mcherry expression levels were investigated using immunofluorescence staining of brainstem slices at the end of the experiment.

**Results:**

Unit recordings could be performed in 12 rats and in a total of 12 neurons (DREADDs: *n* = 7, controls: *n* = 5). Clozapine 0.01 mg/kg did not affect the mean firing rate of recorded LC-neurons; 0.1 mg/kg induced an increased firing rate, irrespective whether neurons were recorded from DREADD or control rats (*p* = 0.006). Co-labeling of LC neurons and mCherry-tag showed that 20.6 ± 2.3% LC neurons expressed the hM3Dq receptor. Aspecific expression of hM3Dq-mCherry was also observed in non-LC neurons (26.0 ± 4.1%).

**Conclusion:**

LC unit recording is feasible in an experimental set-up following manipulations for DREADD induction. A relatively low transduction efficiency of the used AAV was found. In view of this finding, the effect of injected clozapine on LC-NA could not be investigated as a reliable outcome parameter for activation of chemogenetically modified LC neurons. The use of AAV2/7, a vector previously applied successfully to target dopaminergic neurons in the substantia nigra, leads to insufficient chemogenetic modification of the LC compared to transduction with AAV2/9.

## Introduction

The Locus coeruleus (LC)-noradrenaline (NA) pathway is believed to play an important role in the development and treatment of epilepsy (Giorgi et al., 2004). Different preclinical studies demonstrated the anti-epileptic role of endogenous NA since lesioning LC accelerates the rate of amygdala kindling (Giorgi et al., 2004) and attenuates seizure-suppressing effects of Vagus Nerve Stimulation (VNS) (Krahl et al., 1998). In general, it is known that the LC-NA pathway modulates brain excitability which is evident based on hippocampal evoked potentials (EP) and electro-encephalography (EEG). Activation of the LC-NA system induces synaptic plasticity in the form of long term depression or long term potentiation depending on simultaneous activation of the neural target and glutamate release. These paradoxal effects could be explained by the “hot spot theory” referring to the hypothesis that the local increase in NA concentration is depending on the initial activation of the neural target, where local glutamate increases lead to NA increase of LC-varicosities (Lacaille and Harley, 1985; Walling et al., 2004; Brown, 2005; Reid and Harley, 2010; Hansen and Manahan-Vaughan, 2015; Mather et al., 2019).

To further unravel the precise role of the LC-NA pathway in modulation of brain excitability and eventually even in the mechanism of action of VNS, selective LC modulation is desirable. Chemogenetics is a technique able to modulate specific brain structures with high cellular specificity using Designer Receptors Exclusively Activated by Designer Drugs (DREADD). DREADDs are muscarinic metabotropic receptors (hM3Dq for excitation, hM4Di for inhibition) that are genetically engineered to be inert for the endogenous ligand acetylcholine but are known to respond to so called “designer drugs” such as Clozapine-N-Oxide (CNO) (Gomez et al., 2017). CNO is a major metabolite of clozapine, a clinically used antipsychotic drug. Activation of Gq-coupled hM3Dq by CNO was previously shown to activate neurons through phospholipase C (PLC) dependent mechanisms (Alexander et al., 2009). Activation of hM3Dq-NA neurons under isoflurane anesthesia by systemic CNO administration was demonstrated to enhance recovery of arousal from anesthesia by Aston-Jones et al. (Vazey and Aston-Jones, 2014).

Frequently used techniques for *in vivo* transduction of LC neurons consists of an intracerebral injection of a viral vector, such as adeno-associated viral vector (AAV) or canine adeno-associated viral vector (CAV), in the vicinity of LC. To achieve LC specific expression of the DREADD a selective promotor is used such as PRSx8, a synthetic dopamine-β-hydroxylase (DBH) promotor (Hwang et al., 2001; Vazey and Aston-Jones, 2014). Alternatively site-specific recombinase technology can be used which consists of injecting a Cre-dependent AAV vector in TH-Cre transgenic mice. Previous research groups using AAVs mostly used an AAV2/9 which resulted in successful transduction of LC (Vazey and Aston-Jones, 2014; Fortress et al., 2015; Kane et al., 2017; Rorabaugh et al., 2017; Cope et al., 2019; Zerbi et al., 2019).

In this study we examined the feasibility of injecting AAV2/7, carrying the PRSx8-hM3Dq-mCherry construct, to induce LC-specific expression of the excitatory hM3Dq DREADD. To achieve this an AAV2/7 was injected in the rat LC. This AAV serotype was used because it has proven efficiency in transducing high numbers of dopaminergic neurons in the substantia nigra (Van der Perren et al., 2011).

We also aimed to determine the effects of systemic administration of two different subclinical doses of clozapine (0.01 and 0.1 mg/kg). Earlier evidence showed that CNO, upon systemic injection in rats, is back-converted to clozapine (MacLaren et al., 2016). In contrast to CNO, clozapine easily crosses the blood-brain barrier. Since clozapine has high affinity for DREADDs, activation of DREADDs in the brain by systemic CNO is mainly caused by its metabolite clozapine (Gomez et al., 2017). Importantly, at therapeutic doses, i.e., 1–10 mg/kg, clozapine binds to a broad range of neuroreceptors including dopamine D_2__–__4_, serotonin 5-HT_2__*A*_, 5-HT_2__*C*_, muscarinic M_1_, M_2_, M_3_, M_4_, adrenergic α_1_ and α_2_, as well as histamine H_1_ receptors (Svensson et al., 1975). Some of these receptors are expressed in LC (Egan and North, 1985; Szabo and Blier, 2001; Korotkova et al., 2005; Bærentzen et al., 2019).

The aim of our study was to design an experimental approach where selective modulation of neurons of the LC through chemogenetic stimulation would allow to investigate the noradrenergic pathway in the regulation of brain excitability. The aim was to use the hM3DQ DREADD approach, systemic administration of subclinical doses of clozapine and recording of LC neuronal firing modulation with the ultimate aim of performing this in awake epileptic rats in the future.

## Materials and Methods

### Animals

Thirty-two adult male Sprague-Dawley rats (Envigo, The Netherlands) were used in this study and treated according to European guidelines (directive 2010/63/EU). The study protocol was approved by the local Ethical Committee on Animal Experiments of Ghent University (ECD 16/31). Animals were kept under environmentally controlled conditions: 12 h light/dark cycles with artificially dimmed light, temperature and relative humidity at 20–23°C and 40–60%, respectively, with food (Rats and Mice Maintenance, Carfil, Belgium) and water *ad libitum*. All animals were housed individually in type III H cages (Tecniplast, Australia) on wood-based bedding (Carfil, Belgium). Cages were enriched with paper nesting material (Nestil, Carfil, Belgium).

### Viral Vector Administration

Animals (*n* = 32; 9–10 weeks old; 309 ± 15 g body weight) were anesthetized with a mixture of medical oxygen and isoflurane (5% for induction, 2% for maintenance, Isoflo, Zoetis, United Kingdom); body temperature was controlled using a heating pad. Rats were placed in a stereotaxic frame (Stoelting, United States) and the skull was exposed. Bregma was lowered 2 mm relative to lambda (15° head angle) to target the LC and avoid the transverse sinus. Using a Neuro-Syringe (Hamilton model 7001 point style 3, Hamilton company, Nevada, United States) and Quintessential Stereotaxic Injection system (flowrate 2 nl/min, Stoelting, United States), injections of 10 nl of an adeno-associated viral vector (AAV) serotype 2/7 containing a PRSx8-hM3Dq-mCherry plasmid (5.99 × 10^12^ GC/ml) (*n* = 19; 11 unilateral and 8 bilateral) or AAV2/7-PRSx8-eGFP plasmid as a control (*n* = 13; 5 unilateral and 8 bilateral) were performed in the LC (3.9 AP, 1.15 ML relative to lambda, −5.7 DV from dura). After injection the syringe was left in place for an additional 5 min and was then slowly withdrawn to avoid backflow. Following surgery, animals were subcutaneously (s.c.) injected with the non-steroidal anti-inflammatory drug meloxicam (NSAID, 1 mg/kg metacam, Boehringer Ingelheim, Germany) and lidocaine (5% Xylocaine gel, AstraZeneca, United Kingdom) was applied to the incision site to minimize discomfort. All animals recovered 3 weeks in their home cage to allow for optimal viral vector expression (Smith et al., 2016).

### Electrophysiology: *In vivo* Extracellular Unit Recording in Locus Coeruleus Neurons

At least 3 weeks after viral vector injection animals (387 ± 26 g body weight) were used for LC unit recordings. Rats were anesthetized with a mixture of medical oxygen and isoflurane (5% for induction, 1.5% for maintenance, *n* = 18) or were induced with 5% isoflurane followed by an intraperitoneal injection of urethane (1.5 g/kg, *n* = 14). Rats were placed in the stereotaxic frame as described in section “Viral Vector Administration.” For unit recording, a tungsten microelectrode (0.008″/200 μm shank diameter, impedance ≥1.5 MΩ, FHC, United States) was implanted under electrophysiological control and audio monitoring to target LC neurons (Bouret et al., 2003). As a reference and ground, two custom made epidural scalp electrodes, consisting of an insulated copper wire attached to a stainless steel microscrew (1.75 mm diameter; Plastics One, United States) were placed above the left and right frontal cortex. Electrophysiological recordings were amplified (x10 000), filtered (300 Hz-3 kHz) and digitized at 31 kHz using a 1401 micro and Spike2 software (Cambridge Electronic Design, United Kingdom) for online visualization of action potentials and storage for post-processing. Action potentials are detected as input signals crossing a trigger level set by the researcher. LC neurons can be visually identified online as they are characterized by the occurrence of a typical pattern called “a phasic burst inhibition” following a foot pinch (Vazey and Aston-Jones, 2014; Hirschberg et al., 2017). After identification of LC neurons, a stable baseline period of 300 s was recorded followed by subsequent subcutaneous injections of clozapine into the loose skin of the neck, starting with the lowest dose of 0.01 mg/kg and followed by 0.1 mg/kg (dissolved in 3% DMSO in saline). Action potentials were recorded for at least 650 s after each clozapine injection. After the recording period following 0.1 mg/kg clozapine administration, clonidine (0.04 mg/kg), an α2 agonist which inhibits the spontaneous firing of LC-NA neurons, was injected subcutaneously to confirm the LC identity of recorded neurons (Svensson et al., 1975). Recording was stopped when a decrease in the firing frequency at least to baseline levels was observed.

### Histology

At the end of the unit recording sessions, animals were deeply anesthetized with an overdose of sodium pentobarbital (200 mg/kg, i.p.) and transcardially perfused with phosphate-buffered saline (PBS) followed by paraformaldehyde (4%, pH 7.4). The brains were post fixed in paraformaldehyde (4%, pH 7.4) for 24 h and subsequently cryoprotected in a sucrose solution of 10–20–30% at 4°C, snap-frozen in isopentane and stored in liquid nitrogen at −196°C. After 1 h on −20°C, coronal cryosections of 40 μm were made using a cryostat (Leica, Germany). The sections were rinsed twice for 5 min in distilled water (dH_2_O) followed by incubation in 0.5% and 1% H_2_O_2_ for 30 and 60 min, respectively, to block endogenous peroxidase activity. After washing twice for 5 min in PBS, sections were incubated in blocking buffer (BB) made of PBS containing 0.4% Fish Skin Gelatin (FSG) and 0.2% Triton X for 45 min to block non-specific antibody binding sites. The sections were then incubated in primary antibodies to visualize noradrenergic LC neurons and GFP or mCherry tag, respectively, with mouse anti-DBH (1:1000, Merck, clone 4F10.2) and chicken anti-green fluorescent protein (1:1000, Abcam, ab13970) or rabbit anti-red fluorescent protein (targeting mCherry, 1:1000, Rockland, ROCK600-901-379) diluted in BB for 1 h at room temperature and subsequently overnight at 4°C. On the next day the sections were washed twice in blocking buffer for 10 min followed by incubation in secondary antibodies Alexa Fluor goat anti-mouse 488 nm (1:1000, Abcam, Ab 150113) or Alexa Fluor goat anti-mouse 594 nm (1:1000, Abcam, ab150176) against DBH^+^ cells and Alexa goat anti-rabbit 594 nm or Alexa fluor goat-anti chicken 488 nm (against mCherry or GFP tag) diluted in BB for 1 h at room temperature in darkness. After washing twice in PBS for 5 min, a nuclear DAPI staining was performed and subsequent rinsing in PBS (2 × 5 min). Sections were mounted on glass slides and cover slipped using Vectashield H1000 mounting medium (Vector Laboratories, United States) to prevent photo bleaching.

The viral vector expression levels were determined using a fluorescence microscope (Carl Zeiss, Axiovert 200M and Nikon Eclipse TE2000-E, Germany). Pictures of DAPI, DBH and GFP or mCherry were taken with the AxioVision Microscope Software (6D acquisition) connected to the Carl Zeiss fluorescence microscope on the 10x magnification. For each animal that underwent successful unit recording, images were taken from three slices along the anterior-posterior axis of the LC and exported as TIFF files before being analyzed in Fiji-ImageJ software. To determine hM3Dq-DBH colocalization, three sections containing LC per animal were used to quantify the number of DBH^+^ LC cells by placing virtual markers on a merged image of DBH^+^/DAPI. These markers were then copied on the merged image of DBH^+^/mCherry to determine the number of LC cells expressing hM3Dq-mCherry. To quantify aspecific hM3Dq-mCherry expression, cells that were DBH^–^/mCherry^+^ were counted and divided by the total number of mCherry^+^ cells.

### Electrophysiological Data Analysis

Units which responded with a typical phasic bursting/tonic inhibition pattern after foot pinch, of which the activity could be stably recorded upon the administration of both clozapine dosages and whose activity was inhibited by administration of clonidine were included in the analysis. Units were offline identified in Spike2 and confirmed as independent using principal component and autocorrelation (Chaijale et al., 2013). The mean firing frequency per 10 s bins was calculated for an episode of 300 s during baseline (30 data points) and 300 s after injection of both clozapine dosages (30 data points before the next injection covering the maximum effect of each dose). DREADD and control group averages of baseline and clozapine (0.01 and 0.1 mg/kg) epochs were calculated. To determine the effect of group (DREADD vs. control), treatment (baseline, clozapine 0.01 mg/kg and clozapine 0.1 mg/kg) and their interaction a two-way repeated measures ANOVA was performed, followed by *post hoc* Bonferroni test for comparing individual conditions. All data are presented as mean ± standard error of mean (SEM). Statistical analysis was performed in SPSS for windows (version 25). Graphs were made in GraphPad Prism 6.

## Results

### Effect of Systemic Clozapine Administration on LC Firing Frequency

**FIGURE 1:**
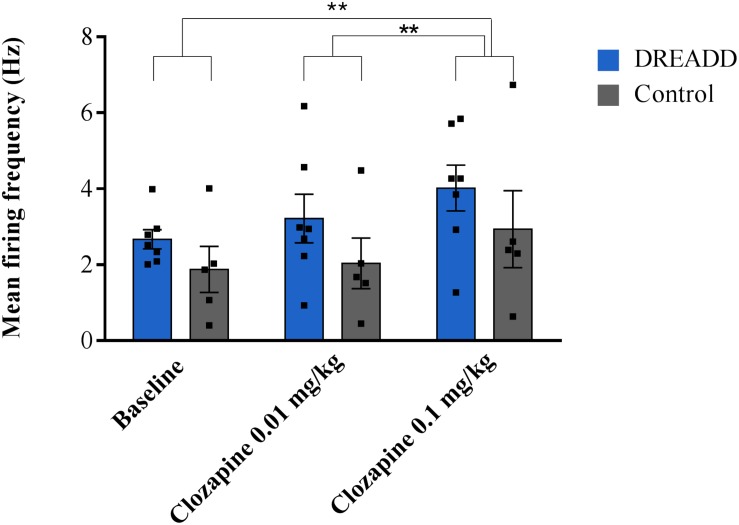
The effect of systemic administration of different doses of clozapine on the mean firing frequency of LC neurons in DREADD and control injected animals. Electrophysiological recordings were performed in 12 animals (DREADD group *n* = 7; control *n* = 5), in each animal one neuron was recorded for a stable baseline period (300 s) followed by subsequent injections of clozapine (0.01 and 0.1 mg/kg, s.c.). No difference in the effect of clozapine on the firing frequency of neurons recorded in DREADD-injected or control animals was observed (*F* = 14.623, *p* = 0.003). The highest dose of clozapine (0.1 mg/kg) increased the firing frequency compared to baseline (*p* = 0.006) whereas the lowest dose of clozapine (0.01 mg/kg) showed no effect (*p* = 0.312). A significant difference between the two doses of clozapine on the mean firing frequency was observed (*p* = 0.003). Each bar represents the mean firing frequency ±SEM (firing frequencies from individual neurons represented by black squares). **p < 0.01 (two way repeated measures ANOVA Bonferroni corrected).

Successful electrophysiological recordings were performed in 12 animals. A total of 12 neurons were recorded, 7 in DREADD-injected animals and 5 in control animals, all characterized by foot pinch elicited phasic bursting and decreased firing frequency after clonidine administration. A two-way repeated measures ANOVA showed no interaction between treatment and group (*F* = 0.212, *p* = 0.655). No significant effect of group was reported (*F* = 1.429, *p* = 0.259) although treatment has a significant effect on the firing frequency (*F* = 14.623, *p* = 0.003). Systemic administration of the 0.01 mg/kg clozapine had no significant effect on the mean firing frequency (2.72 ± 0.48 Hz, *p* = 0.468) of recorded LC neurons compared to baseline (2.34 ± 0.30 Hz) whereas an increased firing rate was observed after 0.1 mg/kg clozapine (3.57 ± 0.54 Hz, *p* = 0.006), irrespective whether recordings were performed in DREADD or control animals. A significant difference between the two doses of clozapine on the firing frequency was observed (*p* = 0.003) (Figure 1). In all LC neurons, firing frequency was reduced to baseline levels or completely inhibited after clonidine administration confirming LC identity.

### Viral Vector Expression

**FIGURE 2:**
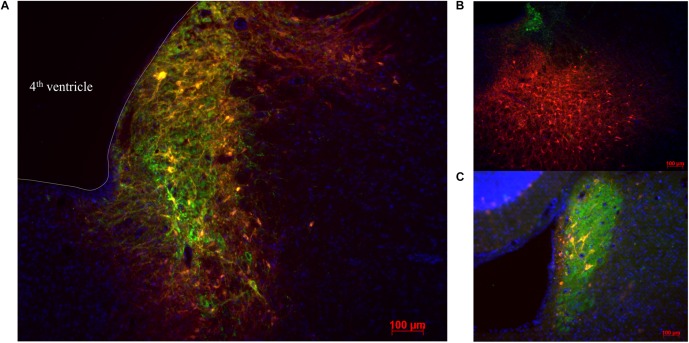
Visualization of hM3Dq-mCherry expression in LC injected with PRSx8-driven AAV. **(A–C)** LC-NA neurons are visualized using primary anti-DBH antibody (green) and expression of hM3Dq DREADD is visualized with the mCherry tag (red). **(B)** Aspecific hM3Dq expression (red) in DBH^-^ non LC neurons. **(C)** Only a low number of transduced neurons are observed in some animals. Scale bar 100 μm.

Double label immunostaining for the mCherry tag coupled to the hM3Dq receptor and DBH was used to quantify the transduction efficiency of the viral vector in LC-NA neurons and its specificity. All animals that underwent successful unit recording at least 3 weeks after viral vector administration showed expression of hM3Dq in LC-NA cells and adjacent axons although there was variability in the fraction of LC cells expressing hM3Dq (Figures 2A–C). HM3Dq expression was evident in 20.6 ± 2.3% LC neurons (21 sections; three sections/animal; range 11–32%). Aspecific expression of hM3Dq-mCherry was also observed in cells outside the LC (26.0 ± 4.1% of mCherry positive cells was DBH negative) (Figure 2B).

## Discussion

In this study we tested the feasibility of an experimental approach where LC neurons were transduced with AAV vectors for induction of DREADD expression, followed by LC unit recording and subcutaneous clozapine injections to investigate the potential to selectively modulate LC-NA neurons. This approach could ultimately be used in the context of investigating the role of the LC-NA pathway in regulating cortical excitability in awake epileptic rats.

For chemogenetic modification we used the adeno-associated viral vector (AAV2/7), previously used in the substantia nigra (Van der Perren et al., 2011), to selectively express the excitatory hM3Dq DREADD in LC neurons using the PRSx8 promoter. Post-mortem analysis of hM3Dq presence in the brainstem revealed low levels of expression in LC in combination with the presence of aspecific expression in surrounding non-LC neurons.

The transduction efficiency of specific cells is defined by the AAV serotype, promotor and injection site (Martin et al., 2002). Our observed transduction levels were in contrast with high specificity and expression levels of >95% in previous studies using AAV2/9-PRSx8-hM3Dq-mCherry to transduce the LC (Vazey and Aston-Jones, 2014). The serotype of the viral vector may be one of the primary reasons for the poor expression levels and aspecificity. The transduction efficiency of an AAV is determined by AAV entry in the target cell (Lai et al., 2002) which is controlled by the AAV capsid with its proteins on the surface that determine the binding specificity and entry of the virion to cells (Rabinowitz et al., 2002; Delbeke et al., 2017). In contrast to other groups who use the AAV2/9 and achieve high expression levels, in this study AAV2/7 was used which means that the genome of serotype 2 is encapsulated in a viral capsid formed by serotype 7 (Vazey and Aston-Jones, 2014; Cope et al., 2019). Probably the presence of different surface proteins presented on the capsid in comparison to AAV2/9 leads to lower binding affinity for the surface receptors on LC-NA cells and possible affinity for non-LC cells, explaining both low expression levels and aspecificity. Although previous research has proven that AAV2/7 is as effective as AAV2/9 in transducing dopaminergic neurons in substantia nigra, this might be different when targeting noradrenergic LC cells due to their different receptors and co-receptors (Lai et al., 2002; Van der Perren et al., 2011).

Next to the AAV serotype, expression specificity is also defined by the promotor. The PRSx8 promoter is a synthetic DBH promoter which consist of eight copies of a promoter sequence from the *cis*-regulatory region of the DBH gene that binds the Phox2a/b transcription factors. This promoter sequence has proven to successfully drive gene expression in catecholaminergic neurons expressing the Phox2a/b transcription factors (Hwang et al., 2001) as described by other groups using AAV2/9-PRSx8 construct observing high expression levels (Vazey and Aston-Jones, 2014). Because of these observations, it is less likely that the observed aspecific expression in our study is due to promotor characteristics. However, it is possible that due to the affinity of the capsid for receptors present on non-LC cells, Phox2 transcription factors present in these non-catecholaminergic cells (Bruinstroop et al., 2012) activate gene expression. Another possible explanation might be the presence of transactivator activity in the AAV inverted terminal repeats. Terminal repeats (TR) contain sequences necessary for replication and packaging of recombinant DNA and cannot be deleted. Haberman et al. (2000) have shown that an AAV TR construct without promoter can initiate gene expression, indicating its transcriptional and promoter ability, resulting in a loss of tissue-specific expression (Haberman et al., 2000). When the number of viral vector virions at the injection site is high [=high multiplicity of infection (MOI)] the amount of TR mediated transcription can increase or promotor leakage can occur, which means that the promotor will be active in non-specific cells, resulting in detectable aspecific expression (Bruinstroop et al., 2012).

Finally we cannot exclude the inadequate delivery of the adeno-associated viral vector in the vicinity of LC as we were not able to localize the exact injection site using post-mortem immunohistochemical analysis (Sara and Hervé-Minvielle, 1995). We use a validated technique to target LC and observe expression in all injected animals supporting the conclusion that the injection was performed correctly (Rorabaugh et al., 2017; Cope et al., 2019).

The ultimate aim of the experiment was to determine whether presumed subtherapeutic doses of clozapine could be used to specifically activate DREADD expressing LC neurons in anesthetized rats.

We found that LC unit recording is feasible following manipulations to induce DREADD expression although the yield is relatively low as we were only able to perform successful electrophysiological recordings in 35% of the animals. This is similar to other research groups targeting locus coeruleus with success rates around 45% and is possibly due to the small size and location of the nucleus in the pontine brainstem (Quinlan et al., 2019). In the current experimental setting, we were not able to draw conclusions on the feasibility to chemogenetically increase LC neuronal activity with clozapine. Since only a limited fraction of LC neurons showed expression of the DREADD upon AAV injection, we could not claim that we recorded from DREADD positive LC neurons in the DREADD group. In fact, this study showed that 0.1 mg/kg clozapine increases the firing rate of LC neurons even in the control group and thus independent of DREADD expression. We can thus conclude that 0.1 mg/kg clozapine does significantly activate non-DREADD neuroreceptors influencing the firing rate of LC neurons. This dose is thus less suitable for future experiments to selectively activate DREADD-expressing LC neurons (Gomez et al., 2017; Mahler and Aston-Jones, 2018).

These DREADD-independent effects of low doses of clozapine were not expected as previous studies using 0.1–10 mg/kg CNO were without effects on LC firing frequency in control animals (Vazey and Aston-Jones, 2014). These doses correspond or are even higher than the doses of clozapine used in this study keeping in mind that 10 mg/kg CNO ∼ 0.1 mg/kg clozapine described by Gomez et al. (2017). A previous study in healthy animals showed an increased firing frequency of LC and ventral tegmental area neurons after intravenous administration of clozapine (0.078 mg/kg – 10 mg/kg) (Nilsson et al., 2005; Schwieler et al., 2008). Pretreatment with a selective antagonist at the glycine site of the NMDA receptor inhibited the activation of LC neurons after administration of the highest dose of clozapine but cannot solely explain the increased firing frequency as administration of a selective partial agonist of the glycine/NMDA receptor did not increase the LC activity. However, it has been shown that clozapine is able to increase extracellular glutamate levels (Daly and Moghaddam, 1993). These findings suggest that a combination of activating the glycine site of the NMDA receptor and increased glutamatergic release, as a result of activation of clozapine sensitive receptors located on glutamatergic afferents to LC, might be responsible for activation of LC noradrenergic neurons after administration of the highest dose of clozapine (Nilsson et al., 2005). Local delivery of clozapine as performed by Vazey et al. (Vazey and Aston-Jones, 2014), instead of systemic administration (s.c.) could possibly rule out major effects of activated glutamatergic afferents to LC, although activation of presynaptic axons would still be possible. However, we chose in our study to subcutaneously inject our animals, knowing that clozapine has proven to cross the blood-brain-barrier easily and to have a high affinity for DREADDs (Gomez et al., 2017). Due to the absence of differences in effects on LC firing frequency after administration of CNO locally or systemically (Vazey and Aston-Jones, 2014) and with the future prospect of performing experiments in awake animals, subcutaneous administration of clozapine was preferred.

Next to activation of endogenous NMDA receptors, recent studies suggest that low doses of clozapine, result in occupancy of endogenous receptors in the brain and significant changes in neurometabolite levels and effects on locomotion, anxiety and cognitive flexibility (Bouret et al., 2003; Smith et al., 2016). Clozapine has affinity for D2, M2, 5-HT1a, α1 and histamine receptors present in LC which can also lead to excitation and increased firing frequency (Egan and North, 1985; Szabo and Blier, 2001; Korotkova et al., 2005; Bærentzen et al., 2019).

From our results and sequence of experimental steps, we conclude that the use of AAV2/7 is less desirable to specifically induce DREADD expression in LC neurons in comparison to previously used approaches. A different AAV serotype such as the AAV2/9 as discussed above leads to higher transduction levels (Vazey and Aston-Jones, 2014; Fortress et al., 2015; Kane et al., 2017; Rorabaugh et al., 2017; Cope et al., 2019; Zerbi et al., 2019). Other groups have even used a different type of viral vector (i.e., CAV) to induce the expression of opsins or engineered ligand gated ion channels in the noradrenergic neurons resulting in high expression patterns after direct injection in LC or administration in LC projection areas (Li et al., 2016; Hirschberg et al., 2017). These more successful approaches of induction of genetic modifications in LC-NA neurons may be due to higher affinity of the virion for the LC-NA surface receptors. An alternative for increasing transduction efficiency and selectivity is the use of a Cre-Lox system for hM3Dq expression in LC (Witten et al., 2011). The group of Harley and Walling et al. used a TH:Cre rat line to induce expression of an opsin in LC by injecting a Cre-dependent AAV with the promoter and gene of interest between loxP sites, assuring selective expression in LC cells that contain Cre recombinase (Quinlan et al., 2019). Because of these findings we believe that for further optimization of the experimental approach studied in this work, transduction efficiency studies with different viral vector serotypes is required when targeting a new brain structure such as a brainstem nucleus, even when a similar type of neurons i.e., noradrenergic neurons are the primary content of the nucleus. An experimental setup with higher transduction efficiency and selectivity is required in combination with a technique to ensure that unit recording was performed in transduced cells to validate the effect of clozapine as a possible selective DREADD ligand. We propose future viral vector experiments with: (1) a different serotype (e.g., AAV2/9 or CAV) to achieve more efficient transduction of LC-NA neurons; (2) a lower titer to avoid aspecific expression due to possible promoter leakage resulting from high MOI and/or (3) higher injected volumes to cover more LC cells (Bruinstroop et al., 2012; Vazey and Aston-Jones, 2014). To allow confirmation on the transduction characteristics of the recorded LC neurons in anesthetized rats juxtacellular labeling with neurobiotin can be performed as proven by other groups performing LC unit recording (Allers and Sharp, 2003; Hajós et al., 2007; Bangasser et al., 2011; Dempsey et al., 2015).

Although this feasibility study demonstrated that our approach is suboptimal to selectively modulate LC, we still believe that chemogenetics, a technique which allows cell-specific modulation is needed to further investigate the role of this brainstem nucleus in brain excitability. A viral vector approach with high transduction efficiency in combination with LC-selective expression of DREADDs and a selective ligand will enable us to study the effect of LC in the epileptic brain and its role in attenuating seizures as observed during VNS (Raedt et al., 2011). So far, experiments that used electrical stimulation or infusion of chemicals to modulate LC function gave ambiguous results concerning its role in modulating hippocampal excitability. Electrical stimulation in LC induced β-receptor-dependent long term depression (LTD) while glutamate injection in LC induced long term potentiation (LTP) in hippocampus (Walling, 2004; Hansen and Manahan-Vaughan, 2015). Some LC activation studies show increased hippocampal responses to afferent input (Haas and Rose, 1987; Dahl and Sarvey, 1990; Sara and Bergis, 1991; Knight and Harley, 2006; Reid and Harley, 2010; Edison and Harley, 2012) while others demonstrate reduced gamma power reflecting decreased activity of local hippocampal neurons (Brown et al., 2005; Walling et al., 2011). These contradictory results might be the result of the small size and deep location of the LC, making the risk of off-target effects by non-selective electrical and chemical stimulation techniques likely.

These findings emphasize that further research using chemogenetics is necessary in the field of LC research, allowing cell-specific modulation and defining its function in brain excitability and confirming its role in the mechanism of action of VNS.

## Data Availability Statement

The raw data supporting the conclusions of this article will be made available by the authors, without undue reservation, to any qualified researcher.

## Ethics Statement

The animal study was reviewed and approved by the Ethical Committee on Animal Experiments of Ghent University.

## Author Contributions

LS, KV, LL, WVL, CG, EC, WW, PB, and RR contributed to the study design and analysis plan. LS and WVL obtained the data. LS, KV, and RR analyzed the data and prepared the manuscript. VB and CV designed and provided the viral vectors. All authors reviewed the manuscript.

## Conflict of Interest

The authors declare that the research was conducted in the absence of any commercial or financial relationships that could be construed as a potential conflict of interest.
